# Analysis of Transcriptome and Terpene Constituents of Scots Pine Genotypes Inherently Resistant or Susceptible to *Heterobasidion annosum*

**DOI:** 10.3389/fpls.2022.947734

**Published:** 2022-07-13

**Authors:** Mengxia Liu, Kai Wang, Matti Haapanen, Rajendra P. Ghimire, Minna Kivimäenpää, Fred O. Asiegbu

**Affiliations:** ^1^Department of Forest Sciences, University of Helsinki, Helsinki, Finland; ^2^College of Forestry, Fujian Agriculture and Forestry University, Fuzhou, China; ^3^Natural Resources Institute Finland (LUKE), Helsinki, Finland; ^4^Department of Environmental and Biological Sciences, University of Eastern Finland, Kuopio, Finland; ^5^Natural Resources Institute Finland (LUKE), Suonenjoki, Finland

**Keywords:** differential expression, monoterpene, pine, resistance breeding, root rot, WGCNA

## Abstract

Root and stem rot caused by *Heterobasidion annosum* is a severe problem in boreal Scots pine. Dissecting the features of disease resistance is generally an essential step in resistance breeding in plants and forest trees. In this study, we explored inherent resistance factors of Scots pine against *H. annosum*. A total of 236 families consisting of 85 full-sib (FS), 35 half-sib population mix (HSpm), and 116 half-sib (HS) families of Scots pine seedlings were inoculated with a *H. annosum* isolate. We sampled needle tissues before inoculation for terpene measurements and RNA sequencing. Based on the lesion area, the extremes of 12 resistant and 12 susceptible families were selected for further analyses. Necrotic lesions resulting from fungal infection were in a weak to moderate relationship with the plant height. Monoterpenes were the principal terpene compounds observed in Scots pine seedlings. Concentrations of 3-carene were significantly higher in pine genotypes inherently resistant compared with susceptible seedlings. By contrast, susceptible genotypes had significantly higher proportions of α-pinene. Gene ontology analysis of differential expressed transcripts (DETs) revealed that response to biotic factors was enriched in resistant seedlings. Functional characterization of individual DETs revealed that higher expression of transcripts involved in response to abiotic stress was common in susceptible genotypes. This observation was supported by the annotation of hub genes in a key module that was significantly correlated with the lesion trait through weighted gene co-expression network analysis (WGCNA) of 16 HS and HSpm samples. These findings contribute to our understanding of constitutive resistance factors of Scots pine against *Heterobasidion* root and stem rot diseases.

## Introduction

The root and stem rot fungi *Heterobasidion parviporum* and *Heterobasidion annosum* within a species complex *Heterobasidion annosum* sensu lato (Fr.) Bref. are necrotrophic pathogens causing severe diseases in forest trees in the boreal region. The preferential host of *H. parviporum* is Norway spruce [*Picea abies* (L.) H. Karst.], whereas *H. annosum* attacks preferentially Scots pine (*Pinus sylvestris* L.) and also other species, including Norway spruce and common juniper (*Juniperus communis* L.) (Müller et al., 2018). Scots pine is among the most common conifer species in Finland. It is estimated that *Heterobasidion* root rot leads to an economic loss of 50 million euros annually in Finland. Abiotic challenges, e.g., climate change is likely to make the situation worse (La Porta et al., 2008). Recently, *Heterobasidion* root rot has been found in pine trees in central Finland, which was previously solely common in southern Finland (Piri et al., 2021). Stump treatment using urea or *Phlebiopsis gigantea* is commonly used to prevent *Heterobasidion* infection (Kärhä et al., 2018). However, the prospect of using resistant genotypes obtained through breeding is a potential and durable approach to manage root and stem rot diseases. Genetic resistance breeding in forest trees is ongoing in some tree species such as *Picea sitchensis* and *Pinus taeda* (Sniezko and Koch, 2017; Naidoo et al., 2019).

Plants including forest trees elicit a series of defense reactions when encountering biotic and abiotic stresses. There are elaborate interactions between biotic and abiotic stress responses. The terminology “cross-tolerance” has recently been used to describe a situation where defense responses induced by one type of stress increase tolerance to other types of stresses (Ramegowda et al., 2020). Nevertheless, abiotic stressors may also result in plant susceptibility to pathogens (Nejat and Mantri, 2017). Chemical defense, including phenolics and terpenes, is a strong defense line for forest trees to fight biotic stress. Terpenes play active roles in plant defense to multiple biotic stresses (Keeling and Bohlmann, 2006; Celedon and Bohlmann, 2019; Toffolatti et al., 2021). For example, (+)-3-carene is a monoterpene constitutively produced in many members of Pinaceae for defense against biotic agents (Fäldt et al., 2003; Hall et al., 2011; Roach et al., 2014). The correlation study of preformed terpene contents in trees and the degree of resistance will aid in a better understanding of their roles in tree defense. Candidate genes in the terpenoid pathway in Scots pine are relevant to *H. annosum* resistance (Mukrimin et al., 2019). Lignin and flavonoid biosynthetic genes together with phenolic and terpene compounds influence root rot resistance (Kovalchuk et al., 2019; Liu et al., 2022). Such findings have shed light on the feasibility and necessity to identify genetic and chemical markers for the prediction of resistant genotypes to improve resistance in conifer trees against root rot diseases.

A transcriptomic analysis offers substantial information on gene expression on a genomic scale and assists researchers in intensely exploring the molecular cues of tree resistance (Kovalchuk et al., 2013; Celedon et al., 2017; Lowe et al., 2017). Thus, conventional methods to test resistant phenotypes, coupled with novel tools such as metabolomics, genomic, and transcriptomic analysis, would deepen our knowledge in tree defense and further accelerate the breeding process. In this study, we sampled needle tissues prior to inoculation and inoculated 236 Scots pine full-siblings and half-siblings with *H. annosum*. Terpene measurements and differential expression analysis coupled with weighted gene co-expression network analysis (WGCNA) using RNA sequencing (RNA-seq) data were carried out to investigate defense mechanisms underlying inherent resistance. Moreover, we analyzed individual transcripts according to their roles in signaling pathways that are central for crosstalk between biotic and abiotic stress responses. We aimed to explore constitutive variation in different resistant phenotypes.

## Materials and Methods

### Plant and Fungal Materials, Needle Sampling, Inoculations, and Measurements

In May 2019, Scots pine seedlings (*ca* 1-year-old, 1,447 seedling plants in total) consisting of 85 full-sib (FS) families, 35 half-sib population mix (HSpm) families, and 116 half-sib (HS) families (Supplementary Table 1) were provided by the Natural Resources Institute Finland (Luke). Each family had six plants, three treated as control (wounds without fungal infection) and three as infected (wounds plus fungal infection). Seedlings were potted using FPM 420 peat substrate (Kekkilä Professional, Finland), and those from different treatments or family types were placed on separate greenhouse tables. Seedlings within the same treatment and family type were randomly arranged. HSpm was originally composed of 50 families from Poland, southern Sweden (Gullabo), southern Finland (Uusikaupunki), central Finland (Kälviä), and northern Finland (Kolari), with 10 families per region. In total, 35 families survived the transfer to the greenhouse, 2, 7, 7, 9, and 10 families from Poland, southern Sweden, southern, central and northern Finland, respectively.

After a 2-month acclimatization in the greenhouse, needle tissues of each plant were sampled before inoculation and stored at −80°C for further use. One *H. annosum* isolate (homokaryon, 03007) previously shown to induce strong necrotic lesions was cultured at 20°C on malt extract–sawdust agar plates (MEA-S) containing 2% Scots pine sawdust, 2% malt extract, and 1.8% agar for 2 weeks before inoculation. In July 2019, inoculations were conducted on the stem at *ca* 15 cm above the greenhouse table according to the following steps: surface sterilization of the stem with 70% ethanol, drilling a hole with the puncher of 2 mm in diameter, and filling the punched hole with MEA-S agar plugs pre-colonized by *H. annosum*. Control treatments received mock inoculation after wounding. Both treatments were sealed with Parafilm M (Heathrow Scientific, USA). At 3 months post-inoculation, the height of each plant was measured from the bottom to the top branches. At 4 months post-inoculation, sapwood lesions of all plants were measured. Lesion size was the sum of vertical and horizontal lesions as described by Keriö et al. (2015). Lesion area was measured using Fiji-ImageJ 1.51 software (Rueden et al., 2017). Lesion area (family mean) of the control and infected plants in each family type were compared by a two-tailed unpaired *t*-test. Lesion area of HSpm infected plants from three regions in Finland was compared using one-way analysis of variance (ANOVA) followed by Tukey's multiple comparisons at 0.05 confidence level. Pearson correlation coefficients and *p* values between lesion size, lesion area, and height based on family means by treatments were computed using the corr. test function in the package psych in R 4.1.0 (R Core Team, 2021; Revelle, 2021).

### Heritability Estimation

Heritability of lesion area was estimated at an individual (${\text{h}}_{\text{HS}}^{2}$, ${\text{h}}_{\text{FS}}^{2}$) and family-mean basis (${\text{h}}_{\text{HSfam}}^{2}$, ${\text{h}}_{\text{FSfam}}^{2}$) separately in the HS and FS data sets using the following equations:


$$\begin{array}{lll} \;h_{HS}^{2}\; & = & \;4\sigma_{GCA}^{2}/(\sigma_{GCA}^{2}+\sigma_{E}^{2})\\ h_{HSfam}^{2}\; & = & \;\sigma_{GCA}^{2}/\left(\sigma_{GCA}^{2}+\left(\sigma_{E/nH}^{2}\right)\right)\\ \;h_{FS}^{2}\; & = & \;4\sigma_{GCA}^{2}/(2\sigma_{GCA}^{2}+\sigma_{SCA}^{2}\;+\;\sigma_{E}^{2})\\ h_{FSfam}^{2}\; & = & \;2\sigma_{GCA}^{2}/\left(2\sigma_{GCA}^{2}+\sigma_{SCA}^{2}\;+\left(\sigma_{E/nH}^{2}\right)\right) \end{array}$$

where $\sigma_{\text{GCA}}^{2}$ is the variance of the general combining ability (GCA), $\sigma_{\text{SCA}}^{2}$ is the variance due to the specific combining ability (SCA), $\sigma_{\text{E}}^{2}$ is the error (residual) variance, and n_H_ is the harmonic mean of the ramets per family (in this case, n_H_ = 6.11 and n_H_ = 5.99 for HS and FS, respectively). The variance components were derived from two mixed models where *Treatment* was a fixed factor and female (HS) or female and male parents (FS) were the random factors. The GCA referred either to the effect of the female parent (HS) or to the average effect of female and male parents (FS). The SCA was an interaction of a specific female–male combination (only estimable from the full-sib data). The analysis was performed using the ASReml software (Gilmour et al., 2015).

### Terpene Analysis

Terpene compounds of needles sampled before inoculation from 12 resistant and 12 susceptible families were quantified as described earlier (Liu et al., 2022). Approximately 100 mg fresh weight of needle tissues was ground in liquid nitrogen and was then extracted in 2 mL of n-hexane at room temperature for 2 h and washed twice with 2 mL n-hexane. The compound 1-chloro-octane (70.1 μg) was used as an internal standard. The extracts were analyzed using a 6890A gas chromatograph (Agilent Technologies, USA) equipped with a HP-5MS UI column (30 m × 0.25 mm × 0.25 μm, Agilent J&W Scientific, USA) and connected to a 5,973 inert mass selective detector (Agilent Technologies, USA). Helium was used as the carrier gas, and linear velocity was about 40 cm/s. The pulsed splitless sampling technique was used with the pulse pressure 25 psi and duration of 0.5 min, and 1 μl was injected. The initial oven setpoint temperature of 50°C was held for 1 min and the column temperature was increased from 50 to 115°C at 5°C/min, then to 280°C at 15°C/min and held for 10 min. Mass numbers from m/z 33 to 350 were recorded. Authentic standards, ChemStation software, as well as Wiley275 and Nist17 libraries were used to identify and quantify monoterpenes and sesquiterpenes. Compounds for which authentic standards were not available were quantified using a compound with a similar chemical structure. For each terpene compound, concentrations μg/mg fresh weight of needle tissues were shown as the mean of 36 resistant or 36 susceptible seedlings (12 resistant and 12 susceptible families, 3 seedlings per family), and the comparison between the two groups was assessed by two-tailed unpaired *t*-test. Pearson correlation coefficients with statistical significances between lesion area and terpene concentrations were computed using the same method as described above.

### Transcriptome Analysis

Total RNA was extracted from *ca* 100 mg of ground needle tissues sampled prior to the inoculation using the method previously described by Chang et al. (1993). The ground sample was transferred to a sterile 2 ml Eppendorf tube, and 900 μl extraction buffer (preheated at 65°C) and 9 μl DTT (1 mol/L) were added. The extraction buffer contained 2% CTAB, 4% PVP (K30), 100 mM Tris-HCl, 25 mM EDTA, and 2 M NaCl. The mixture was vortexed and incubated at 65°C for 15 min. Equal volumes of chloroform: isoamyl alcohol (24:1) were added, followed by mixing well, and centrifugation at 10,000 g at room temperature for 10 min. The upper phase was transferred to a new 2 ml Eppendorf tube and then equal volumes of chloroform: isoamyl alcohol (24:1) were added and the above step was repeated. To precipitate RNA, 1/4 volume of 10M LiCl (42.4 g/mol) was added, mixed well, and kept at 4°C overnight. The sample was centrifuged at 10,000 g at 4°C for 30 min. The supernatant was pipetted out and the pellet was washed by adding 100 μl cold 70% ethanol. The centrifugation was repeated for 5 min. The supernatant was pipetted out and the pellet was dried in the sterile hood. The pellet was resuspended in 10–20 μl nuclease-free water. Extracted RNA was quantified by NanoDrop 2000c (Thermo Fisher Scientific, USA) and quality checked with Agilent 2100 bioanalyzer (Agilent Technologies, Germany). Equal amounts (1 μg) of total RNA from three seedlings per family were pooled together as one sample. Samples of 12 resistant and 12 susceptible families (36 resistant and 36 susceptible seedlings) for terpene detection were also used for RNA-seq. RNA-seq was performed with paired-end (150 bp) sequencing using an Illumina NovaSeq 6000 platform at Novogene (UK). RNA-seq reads for 24 samples are available in the NCBI GEO database with the accession number GSE200311. SortMeRNA v4.2.0 was used to remove ribosomal RNAs from raw reads (Kopylova et al., 2012), followed by the trimming of low-quality reads at Q < 20 in 5-base sliding windows and adaptor sequences by Trimmomatic v0.39 with a minimum length of 50 bp (Bolger et al., 2014). Trimmed reads were qualified by FastQC v0.11.8 and MultiQC v1.8. *De novo* transcriptome assembly was constructed with trinity 2.11.0 (Grabherr et al., 2011), with setting normalize max read cov 50 and min kmer cov 2. A trinity assembly with a size of 288 Mb was produced with GC content 42.08% and E90N50 value 1,824 bp. Sample-specific alignment was conducted with bowtie2 (Langmead and Salzberg, 2012). Alignment-based quantification was performed with RNA-Seq expression estimation by Expectation-Maximization (RSEM) (Li and Dewey, 2011).

Differential expression analysis for each family type was carried out using the package DESeq2 in R (Love et al., 2014). Transcripts that were expressed over one count in at least four libraries were kept. Transformed data by variance stabilizing transformation (VST) were input to perform principal component analysis (PCA). To produce a list of differentially expressed transcripts (DETs), cut-offs were set as Benjamini-Hochberg adjusted *p* value (FDR) < 0.05 and fold change (FC) > 2. Gene ontology (GO) enrichment of DETs was analyzed using the R package clusterProfiler (Wu et al., 2021). GO terms of all Trinity assembly transcripts were annotated through Blast2GO (Conesa et al., 2005). The description file of all GO terms (go-basic.obo) was downloaded from the Gene Ontology database. Significantly enriched terms (FDR < 0.05) were eliminated due to gene overlaps using the function reduce_overlap in the package GOplot in R (Walter et al., 2015). The R package WGCNA was used for co-expression network analysis of HS and HSpm samples (Langfelder and Horvath, 2008). Lowly expressed transcripts with count <1 in all libraries and count <100 in at least eight libraries were removed followed by VST transformation. Gene modules were detected using 1-step network construction function blockwiseModules with proper soft-thresholding power, minimum module size of 100, and merging threshold function of 0.25. Hub genes in the key module were identified using cut-offs of eigengene-based connectivity (KME) ≥ 0.8 and geneTraitSignificance ≥ 0.2.

## Results

### Necrotic Lesions and Correlations With Plant Height

**Figure 1:**
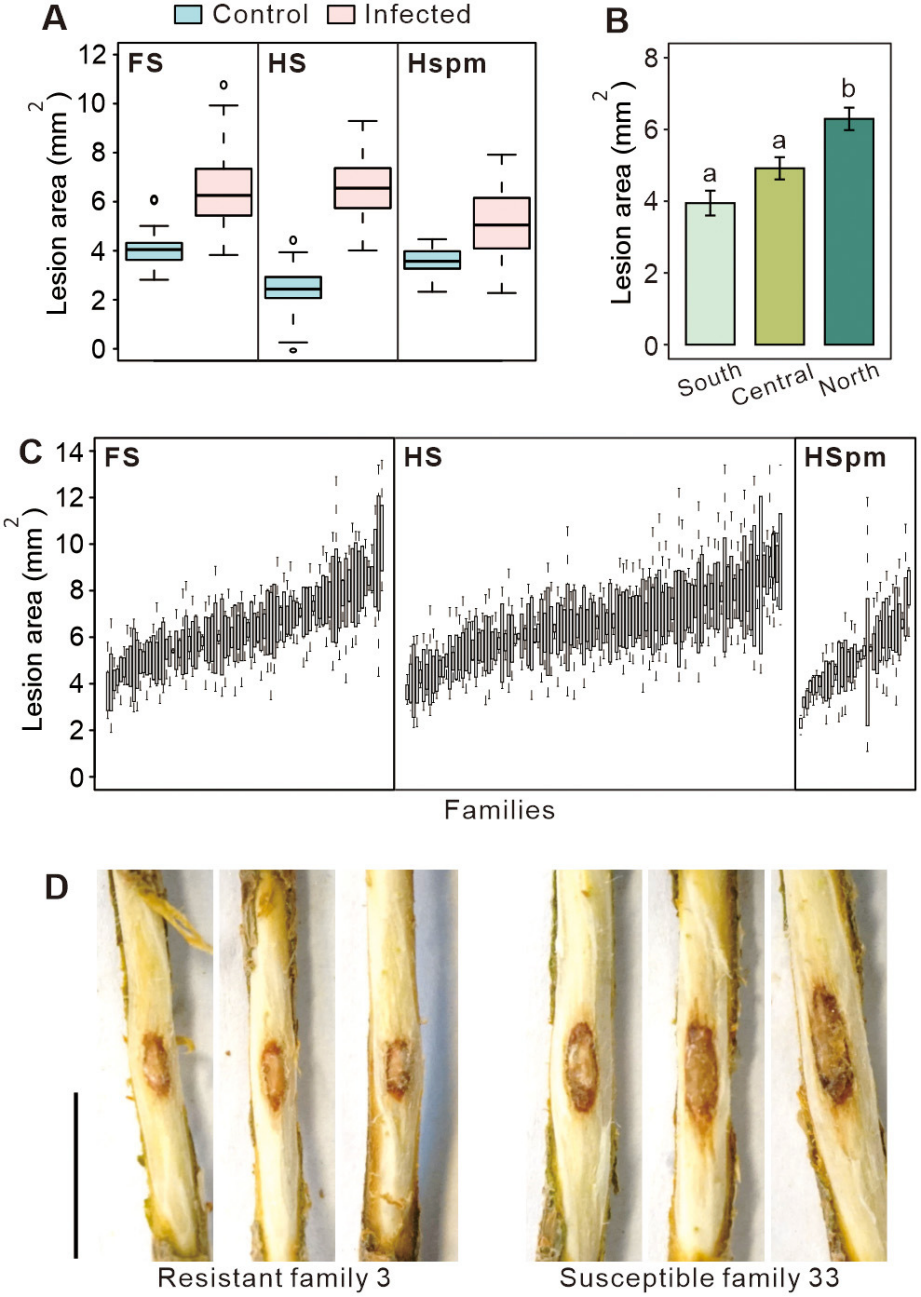
Lesion measurements of Scots pine (*Pinus sylvestris* L.) seedlings treated as control and *Heterobasidion annosum*-infected. **(A)** Boxplots of lesion area of control and infected samples (FS, full-sib, *n* = 85; HS, half-sib, *n* = 116; HSpm, half-sib population mix, *n* = 35). **(B)** Means of lesion area of infected HSpm families from the south (family 293-302, *n* = 7), central (family 283-292, *n* = 9), and north (family 303-312, *n* = 10) of Finland. Error bars represented the standard error of the mean. Statistical significances were marked with distinct letters above the error bars using one-way ANOVA followed by Turkey's multiple comparisons at the 0.05 confidence level. **(C)** Boxplots of family-mean lesion area of infected FS, HS, and HSpm seedlings. **(D)** Necrotic lesions were observed in the sapwood in a resistant family (FS family 3) and a susceptible family (FS family 33). Black lines were the scale bars of 10 mm.

**Figure 2:**
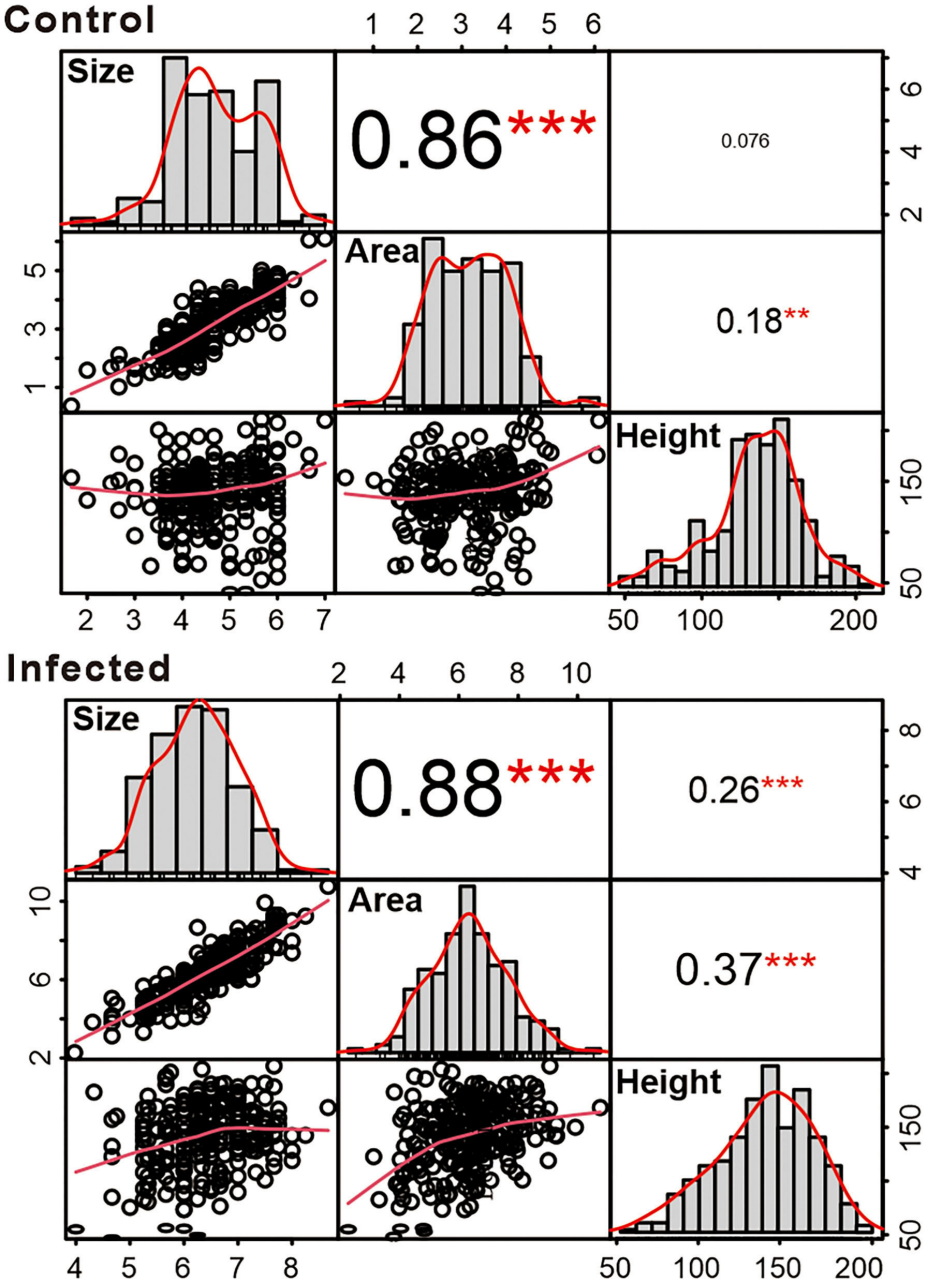
Correlation matrix plot between lesion size, lesion area, and height of Scots pine (*Pinus sylvestris* L.) families treated as control and *H. annosum*-infected (*n* = 236). Bivariate scatterplots with a fitted smooth line were under the diagonal. Pearson correlation coefficients with significance levels were above the diagonal and asterisks indicated the significant differences (*p < 0.05, **p < 0.01, ***p < 0.001).

Necrotic lesions observed in the stem sapwood caused by *H. annosum* infection were larger than those of the control samples (Figure 1A and Supplementary Table 2). The average lesion area of control and infected seedlings was 3.2 mm^2^ (SE 0.07 mm^2^) and 6.3 mm^2^ (SE 0.09 mm^2^), respectively, with significant differences at *p* < 0.001 for the comparison between the two treatments in each family type. Variations in lesion size, lesion area, and plant height were detected among families and data of the above traits were normally distributed (Figure 2). Lesion area of infected samples ranged from 3.8 to 10.8, 4.0 to 9.3, and 2.3 to 7.9 mm^2^ for FS, HS, and HSpm, respectively (Figure 1C and Table 1). Lesion size was strongly correlated with lesion area. Plant height showed a weak to moderate relationship with lesion area, of which coefficients were 0.18 and 0.37 for control and infected samples, respectively (Figure 2). Individual heritability estimates of lesion area of FS and HS were similar, around 0.35, whereas FS represented a larger value on the family-mean basis at 0.53 than HS at 0.38 (Table 2).

**Table 1:** Lesion area (mean and standard error, SE) of resistant and susceptible Scots pine (*Pinus sylvestris* L.) families after being infected by *H. annosum* (*n* = 3).

**Family type**	**Group**	**Family**	**Lesion area (mm** ^**2**^ **)**	
			**Mean**	**SE**
FS	Resistant	86	3.83	1.00
	Resistant	83	4.15	1.42
	Resistant	3	4.17	0.50
	Resistant	59	4.28	0.25
	Susceptible	13	8.98	0.66
	Susceptible	33	9.30	1.10
	Susceptible	5	9.93	1.91
	Susceptible	10	10.76	1.66
HS	Resistant	111	4.01	0.38
	Resistant	184	4.03	0.57
	Resistant	157	4.12	0.93
	Resistant	134	4.35	0.72
	Susceptible	139	9.04	0.95
	Susceptible	202	9.07	0.92
	Susceptible	168	9.25	1.49
	Susceptible	165	9.29	1.14
HSpm	Resistant	302	2.28	0.25
	Resistant	275	3.13	0.29
	Resistant	296	3.32	0.25
	Resistant	300	3.90	0.29
	Susceptible	308	6.61	1.38
	Susceptible	311	6.63	0.22
	Susceptible	303	7.65	1.60
	Susceptible	307	7.92	0.94

**Table 2:** Variances of the general and specific combining effects (GCA, SCA) and heritability of lesion area in the HS (half-sib) and FS (full-sib) data of Scots pine (*Pinus sylvestris* L.) seedlings treated as control and *H. annosum*-infected.

**Family type**	**σ^2^GCA**	**σ^2^SCA**	**σ^2^E**	**h^2^HS, h^2^FS**	**h^2^HSfam, h^2^FSfam**
HS	0.26	N/A	2.53	0.37 (0.12)	0.38 (0.08)
FS	0.19	0	2.03	0.32 (0.16)	0.53 (0.08)

*Standard errors are shown in parentheses*.

Seedlings were considered to be resistant if they had lesion areas of 2.3–4.4 mm^2^ while susceptible seedlings had a lesion area of 6.6–9.9 mm^2^. Based on the family-mean lesion area, 12 resistant and 12 susceptible families of FS, HS, and HSpm were selected for further terpene and RNA-seq analyses (Table 1). Lesions displayed obvious differences between resistant and susceptible seedlings (Figure 1D). Compared with FS and HS, HSpm seedlings showed a smaller lesion area. Families 302, 296, and 300 in HSpm from southern Finland showed enhanced resistance, whereas 307, 303, 311, and 308 from the north were mostly susceptible. Means of lesion area of infected samples increased from the south (3.9 mm^2^) to central (4.9 mm^2^) and north (6.3 mm^2^) parts of Finland (Figure 1B and Supplementary Table 3).

### Differences in Monoterpene Contents Between Resistant and Susceptible Seedlings

From needle tissues of 36 resistant and 36 susceptible seedlings which were sampled prior to inoculation, 17 monoterpene and 12 sesquiterpene compounds were quantified (Supplementary Table 4). Monoterpenes were the predominant terpene compounds, of which total concentration was *ca* 20-fold higher than sesquiterpenes. α-Pinene and β-caryophyllene showed the highest concentrations among monoterpenes and sesquiterpenes, respectively. A few monoterpenes were significantly different between resistant and susceptible seedlings (Table 3). 3-Carene was highly abundant in resistant genotypes compared to susceptible ones. By contrast, the susceptible group had highly concentrated tricyclene, β-pinene, camphene, limonene+β-phellandrene, p-cymene, and α-pinene. Contents of these compounds were positively highly correlated with each other but negatively correlated with 3-carene (Supplementary Table 5). Correspondingly, lesion area was negatively correlated with 3-carene content with a coefficient at −0.34, but positively with tricyclene and β-pinene at 0.28 and 0.26, respectively.

**Table 3:** Monoterpene concentrations (means and standard error, SE) of resistant and susceptible Scots pine (*Pinus sylvestris* L.) seedlings and their Pearson correlation coefficients (*r*) with lesion area (*n* = 36).

**Compound**	**Pearson correlation**		**Concentration (μg/mg fresh weight)**				
			**Resistant**		**Susceptible**		***p-value***
	***r***	***p***	**Mean**	**SE**	**Mean**	**SE**	
Bornyl acetate	0.04	0.768	1.19	0.22	1.94	0.41	0.115
Tricyclene	0.28	**0.016**	2.13	0.17	3.40	0.37	**0.003**
trans-β-Ocimene	−0.19	0.119	3.53	0.41	3.09	0.45	0.474
Sabinene	−0.07	0.587	3.83	0.47	5.21	0.90	0.177
β-Pinene	0.26	**0.027**	6.28	0.77	9.37	0.99	**0.016**
Myrcene	−0.10	0.402	7.03	0.60	7.01	0.50	0.973
Camphene	0.22	0.065	8.45	0.66	11.79	1.05	**0.009**
Limonene + β-Phellandrene	0.19	0.116	8.73	0.54	11.37	1.01	**0.025**
Terpinolene	−0.30	**0.010**	11.16	1.22	8.23	1.11	0.079
p-Cymene	0.14	0.245	15.63	1.17	20.98	2.11	**0.030**
3-Carene	−0.34	**0.003**	133.95	15.27	90.07	13.31	**0.034**
α-Pinene	0.16	0.167	148.32	11.33	203.51	17.61	**0.010**

*Statistical analysis was assessed with two-tailed unpaired t-test*.

*Only the compounds with the concentration of over 1.0 μg/mg fresh weight were listed*.

*P values lower than 0.05 were bold*.

### Highly Expressed Transcripts in Resistant or Susceptible Families

**Figure 3:**
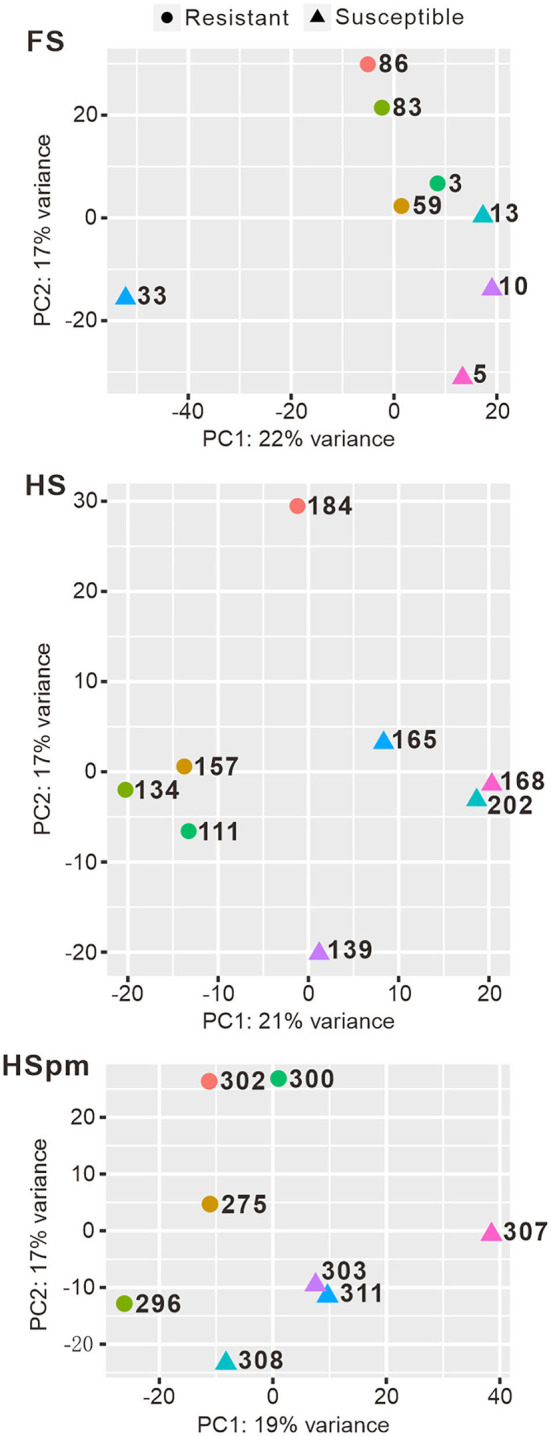
Principal component analysis (PCA) of the expression level of transcripts in resistant and susceptible Scots pine (*Pinus sylvestris* L.) seedlings.

Each library obtained 37–54 million paired-end reads, which were *de novo*-assembled through the Trinity platform with alignment rates of 91.4–98.9%. Although we mapped the reads against an alternative reference genome of *Pinus taeda* (Pyhäjärvi et al., 2020), the mapping rates were very low for each library. Samples differing in resistance to *H. annosum* were separated along the y-axis, x-axis, and the diagonal for FS, HS, and HSpm, respectively, in PCA plots (Figure 3).

**Figure 4:**
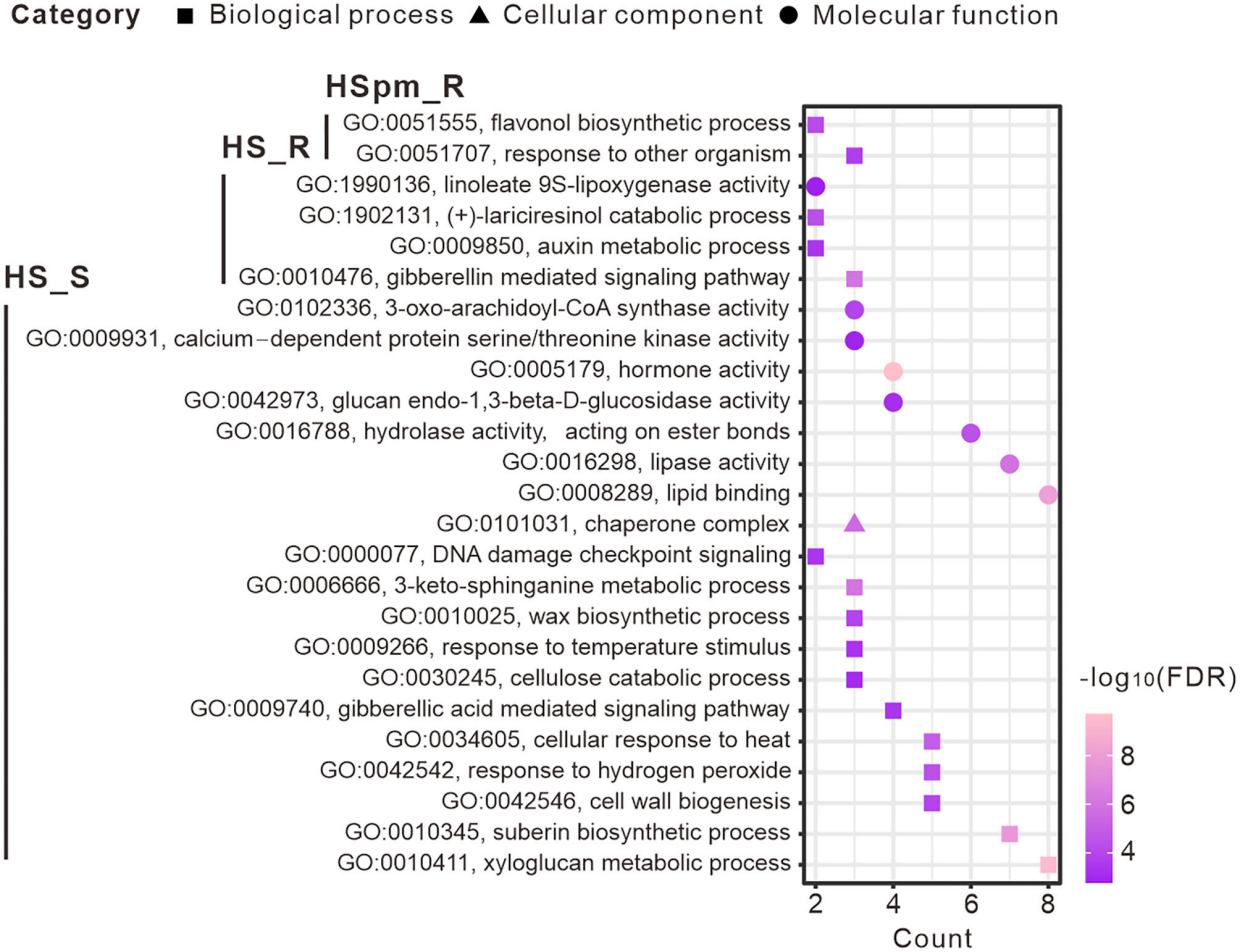
Enriched GO terms of DETs under biological process, cellular component, and molecular function.

Differential expression analysis of each family type identified transcripts that were highly expressed either in resistant or susceptible seedlings (Supplementary Table 6). Functional analysis of subsets of DETs showed two significantly enriched GO terms in HSpm and 23 terms in HS (Figure 4). Resistant samples of HSpm were enriched in DETs involved in the flavonol biosynthetic process and response to other organisms. While HS-resistant samples were enriched in auxin metabolic process and linoleate 9S-lipoxygenase activity, other terms such as hormone activity, cellular response to heat, and wax biosynthetic process were mostly prevalent in susceptible genotypes.

**Figure 5:**
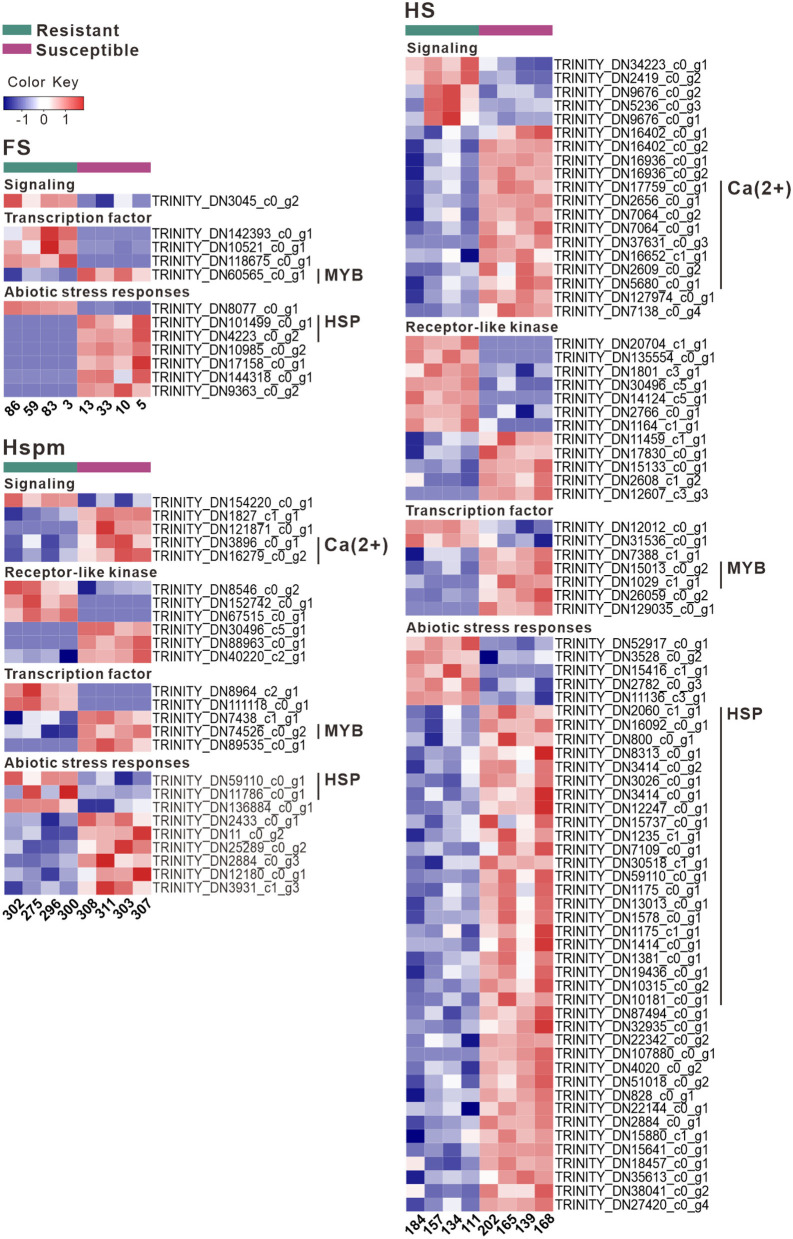
Heatmap of DETs associated with signaling pathways and abiotic stress responses.

Among DETs of each family type, 36–57% were annotated (Supplementary Tables 6, 7). Signaling was primarily mediated by plant hormones such as jasmonic acid, abscisic acid, and gibberellins (Figure 5). Calcineurin B-like (CBL) interacting-protein kinases (CIPKs), RALF-like proteins (RALFLs), and calcium-dependent protein kinases (CDPKs) in susceptible seedlings could play a role to regulate calcium-dependent signaling. Despite differences in resistant capability against *H. annosum*, all samples had highly expressed transcripts involved in defense encoding disease resistance proteins, pathogenesis-related proteins, and key enzymes in the phenylpropanoid and terpenoid biosynthesis. Susceptible samples showed increases in DETs such as MYB transcription factors (MYBs) and triphosphate tunnel metalloenzyme 2 (TTM2), as well as abundant DETs associated with abiotic stress responses (Figure 5). Susceptible samples of HS had increased levels of 22 transcripts encoding heat shock proteins (HSPs), but negligible transcript levels were observed in other samples.

**Figure 6:**
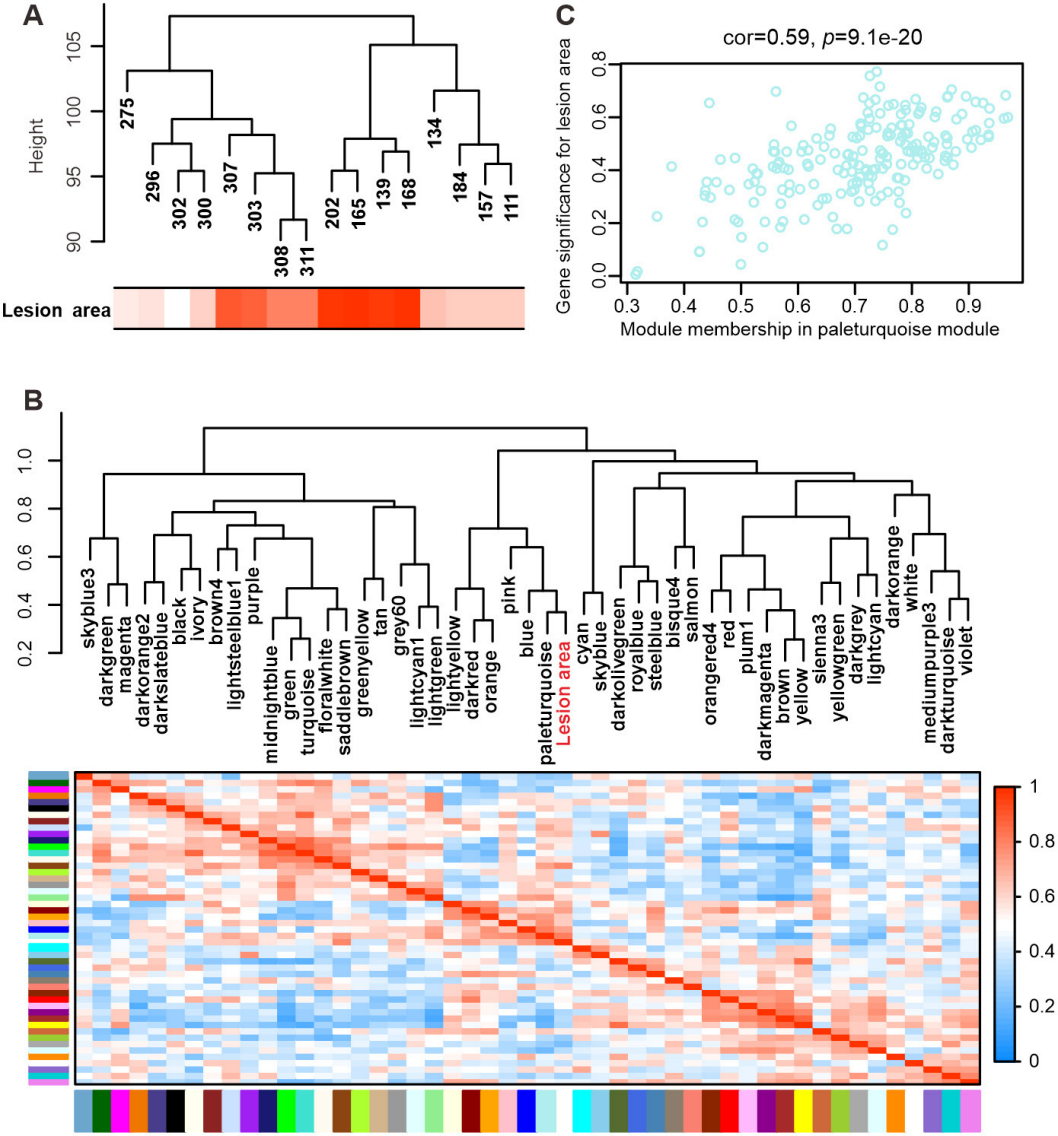
Weighted gene co-expression network analysis (WGCNA) of HS (half-sib) and HSpm (half-sib population mix) samples. **(A)** Clustering dendrogram of samples based on their Euclidean distance and the heatmap of lesion area. **(B)** Visualization of the eigengene network representing the relationships among modules and lesion area. Panel (above) shows a hierarchical clustering dendrogram of the eigengenes. Panel (below) displays a heatmap of the eigengene adjacency. **(C)** Scatterplot gene significance for lesion area vs. module membership in the paleturquoise module.

In addition to functional characterization of DETs, we applied WGCNA to shed light on co-expression networks between transcripts according to their common expression trends across all half-sib (HS and HSpm) samples. After filtering, 20,618 transcripts were used to construct the network to find key modules of highly correlated genes. Samples from the same family type, having a similar lesion area clustered well (Figure 6A), where red means a large lesion and white a small lesion. Using the soft-thresholding power of 6, we identified 49 modules (Supplementary Figures 1A,B). Module size ranged from 123 to 2,448 transcripts. There were 957 transcripts in the gray module that failed to cluster to any module. The dark gray, saddle brown, and pale turquoise modules were significantly correlated with lesion area with coefficients at −0.53, 0.59, and 0.63, respectively (Supplementary Figure 1C). We considered the pale turquoise as the key module since it was closely clustered with the lesion area (Figure 6B). A total of 196 transcripts in the module were highly correlated with the lesion area (Figure 6C). Among them, 72 transcripts were hub genes with high connectivity, and 58 of them were annotated with 20 transcripts for HSPs (Supplementary Table 8), mostly enhanced in the susceptible genotypes. Hub genes are transcripts with high intramodular connectivity, which tend to be strongly correlated with the lesion trait.

## Discussion

Lesion area, instead of lesion size, was used to evaluate resistant phenotypes, which further highlights the differences in response to fungal infection, especially for small-sized plants (Liu et al., 2022). We reasoned that lesion area could be a more accurate method to evaluate resistance partly based on the dichotomous branching and mycelia nature of fungal morphology during invasive growth. Variation in lesion area among families indicated that susceptibility to *H. annosum* varied among genotypes. Lesions observed in wounded control samples implied that defense responses could be triggered by mock inoculation (Lim et al., 2021). Moderate estimates of heritability for lesion area in FS and HS suggested that the lesion trait was influenced by host genetics. Similar results regarding disease resistance in Scots pine have been reported. Narrow-sense heritability estimates of 0.38–0.75 for Dothistroma needle blight severity differed between half-siblings and full-siblings (Perry et al., 2016). A narrow-sense heritability of 0.37 for root rot resistance was higher than those for many growth traits (Rieksts-Riekstinš et al., 2020), and the high estimates were documented to be valuable for breeding (Marčiulynas et al., 2020). A positive relationship between height and lesion area indicated that seedlings of larger size were much more susceptible to *H. annosum*. These results were consistent with a previous study by Mukrimin et al. (2019). They reported a significant correlation of 0.53 between stem diameter and phloem vertical lesion length of Scots pine seedlings at 10–15 years old resulting from *H. annosum* infection, but a negligible value for the mock control. In Norway spruce (17 years old), growth traits including diameter and height were positively correlated with lesion length in the inner bark and fungal growth (Swedjemark and Karlsson, 2004). Results with respect to phenotyping measurements suggested that resistance to *H. annosum* in Scots pine seedlings was under genetic control and supported the growth and defense trade-off hypothesis, where bigger seedlings are susceptible to fungal infection with larger lesions.

Monoterpenes, as major constituents of oleoresin, play essential roles in the physical and chemical defense of conifer trees (Kovalchuk et al., 2013). Similar to our observations, 3-carene and α-pinene were the most abundant terpene compounds in needles of Scots pine and whitebark pine trees either under healthy conditions or infected by fungal pathogens (Bullington et al., 2018; Mukrimin et al., 2019). Several highly concentrated monoterpenes were detected in tree genotypes susceptible to *H. annosum*. Terpenoid biosynthesis was documented to be enriched in Masson pine that was susceptible to pine wood nematode compared to resistant-genotypes (Liu et al., 2017). Mukrimin et al. (2019) found that the expression of α-pinene synthase in Scots pine had a positive relationship with lesion size. By contrast, increased monoterpene contents (α-pinene, tricyclene, and α-longipinene) were observed in asymptomatic (resistant) Norway spruce genotypes infected with *Heterobasidion* (Kovalchuk et al., 2019; Liu et al., 2022). Two subspecies of Scots pine showed different terpene concentrations and proportions (Achotegui-Castells et al., 2013). Variation in terpene concentrations between the samples of different susceptibility demonstrated constitutive genetic differences in resistance. Other authors have noted that the monoterpene amount increases in resistant trees after the induction of defense responses, but remains unchanged in susceptible trees (Hall et al., 2011). Thus, monoterpene contents merit to be further explored as a chemical marker for resistance or susceptibility. In our study, 3-carene was the only compound showing a negative and moderate correlation with lesion area. Notably, Scots pine trees in the south of Finland had lower α-pinene and higher 3-carene contents, but the opposite composition for the trees from the north (Muona et al., 1986; Manninen et al., 2002). In this study, smaller lesions in seedlings from southern Finland with enhanced resistance were observed. It is possible that the increased resistance could be due to the high 3-carene concentration prevalent in seedlings from southern Finland compared to northern Finland.

According to the transcriptome analysis, resistant and susceptible seedlings had comparable amounts of DETs involved in signaling pathways. Susceptible seedlings particularly in the HS family type showed a predominantly larger number of DETs associated with abiotic stress responses, which was in line with our previous findings (Liu et al., 2022). The WGCNA study displayed a large proportion at 34.5% of hub genes for HSPs, which might contribute to enhanced seedling susceptibility to *H. annosum*. Mechanisms governing abiotic stress tolerance have been shown to have both synergistic and antagonistic effects on disease resistance (Kissoudis et al., 2014). Dissecting the interaction between stress signaling pathways can aid in understanding these different effects.

MYB transcription factors are a group of components in the modulation of stress signal transduction, regulating stress responses such as the biosynthesis of the cell wall and secondary metabolites related to defense. *PtMYB1, PtMYB4*, and *PtMYB8* are involved in the phenylpropanoid pathway in loblolly pine, and *PtMYB14* induces the sesquiterpene production in conifer trees (Bedon et al., 2010). According to the review by Ramegowda et al. (2020), OsMYB4, MYB96, and PacMYBA confer tolerance to abiotic stress and resistance against pathogens in Arabidopsis, where ABA-SA signaling networks had influence. Members of various MYB families can positively and negatively regulate cold stress responses in Arabidopsis and Korean pine (Wang et al., 2020).

Heat shock triggers the production of a group of low molecular weight proteins, HSPs in spruce and pine seedlings (Gifford and Taleisnik, 1994). As molecular chaperones, HSPs have been considered as stress proteins, enabling the activation and stabilization of signaling proteins including hormone receptors, transcription factors (TFs), and kinases (Xiang et al., 2018). The main functions of HSPs were summarized by Wang et al. (2004), with roles in protein folding, assembly, translocation, and degradation; the stabilization of proteins and membranes; and protein refolding under stress conditions. Most HSPs detected in this study belonged to the small HSP family with a molecular mass of around 20 kDa. Small HSPs in plants respond to multiple environmental stressors such as heat, cold, drought, salinity stress, and their accumulation is promisingly useful to improve tolerance to stress (Wang et al., 2004; Xiang et al., 2018). Even though roles of HSPs relevant to stress tolerance have not been much studied in conifer trees compared with crop plants, a real-time PCR study of loblolly pine revealed that *HSP* genes can be induced by drought stress (Vásquez-Robinet et al., 2010), and seasonal variation in the HSP content in needles of Scots pine assists plants to adapt to temperature changes all year round (Korotaeva et al., 2012).

According to GO analysis, resistant seedlings evoke a variety of responses to repel a fungal attack, for instance, lipoxygenase (LOX) together with the ethylene response factor (ERF) and the TF function in response to necrotrophs (Bürger and Chory, 2019). Transcripts associated with Ca^2+^-mediated signaling, MYBs and HSPs, represented higher abundance in susceptible seedlings, which suggests that the activation of signaling pathways in abiotic stress might enhance susceptibility to root rot. Susceptible seedlings had other DETs encoding defense proteins (Supplementary Table 7). A negative regulator of SA-signaling, TTM2, has a negative effect on the defense response to pathogens (Ung et al., 2014). EDS1 cooperating with PAD4 triggers a hypersensitive response (HR) that cannot control the invasive growth of necrotrophic pathogens, consequently resulting in larger lesions (Feys et al., 2001). Protein EXORDIUM in the brassino-steroid-dependent regulation of growth-defense trade-offs can promote growth but suppress disease resistance (Coll-Garcia et al., 2004; Bürger and Chory, 2019), which is in agreement with positive correlations between height and lesion area in our study. Besides, it appears that seedlings from northern Finland showed enhanced tolerance to environmental challenges during transportation and re-potting, whereas they were more susceptible to infection. The results further lend credence to the fact that seedlings with tolerance to abiotic stress seem to be more susceptible to *H. annosum*.

In this study, we selected a set of candidate resistant and susceptible full-sib and half-sib families. The resistance ability of their mother trees can be tested further, which will provide promisingly valuable plant materials for genetic breeding programs in Scots pine. We resorted to chemical and transcriptome analysis to understand the inherent resistance to *H. annosum* in Scots pine. The results revealed that resistant genotypes had higher contents of 3-carene, whereas susceptible genotypes had higher concentrations of α-pinene. Terpene composition, especially for α-pinene and 3-carene, also seems to be influenced by the geographical origin of the Scots pine families. Additionally, transcripts associated with abiotic and biotic response interactions were found to be relevant to increased susceptibility of the seedlings. Compared with our previous work on Norway spruce (Liu et al., 2022), the results revealed some commonality and differences in the metabolic pathway for disease resistance in these two conifer species. Overall, our findings indicate that the approach of sampling needle tissue prior to destructive inoculation may have a potential merit for speeding up the large-scale disease resistance screening work.

## Data Availability Statement

The datasets presented in this study can be found in online repositories. The names of the repository/repositories and accession number(s) can be found in the article/Supplementary Material.

## Author Contributions

FA and ML designed the study. ML performed greenhouse experiments and extracted RNA. MH estimated the heritability. RG and MK conducted terpene detection. ML and KW analyzed the data and drafted the manuscript. FA conceived the study and helped to draft the manuscript. All authors edited and revised the manuscript.

## Funding

The study was supported by the research funding from Academy of Finland (Grant No. 307580) to FA, scholarship from China Scholarship Council (Grant No. 201607960002), and Niemi Foundation (Grant No. 20200006) to ML.

## Conflict of Interest

The authors declare that the research was conducted in the absence of any commercial or financial relationships that could be construed as a potential conflict of interest.

## Publisher's Note

All claims expressed in this article are solely those of the authors and do not necessarily represent those of their affiliated organizations, or those of the publisher, the editors and the reviewers. Any product that may be evaluated in this article, or claim that may be made by its manufacturer, is not guaranteed or endorsed by the publisher.
