# Supplementary material for: Analysis of Transcriptome and Terpene Constituents of Scots Pine Genotypes Inherently Resistant or Susceptible to Heterobasidion annosum
Source: Front Plant Sci. 2022 Jul 13;13:947734. doi: 10.3389/fpls.2022.947734 (PMC9326466; doi:10.3389/fpls.2022.947734)
Supplement: Supplementary file 2 [file Image_1.PDF]

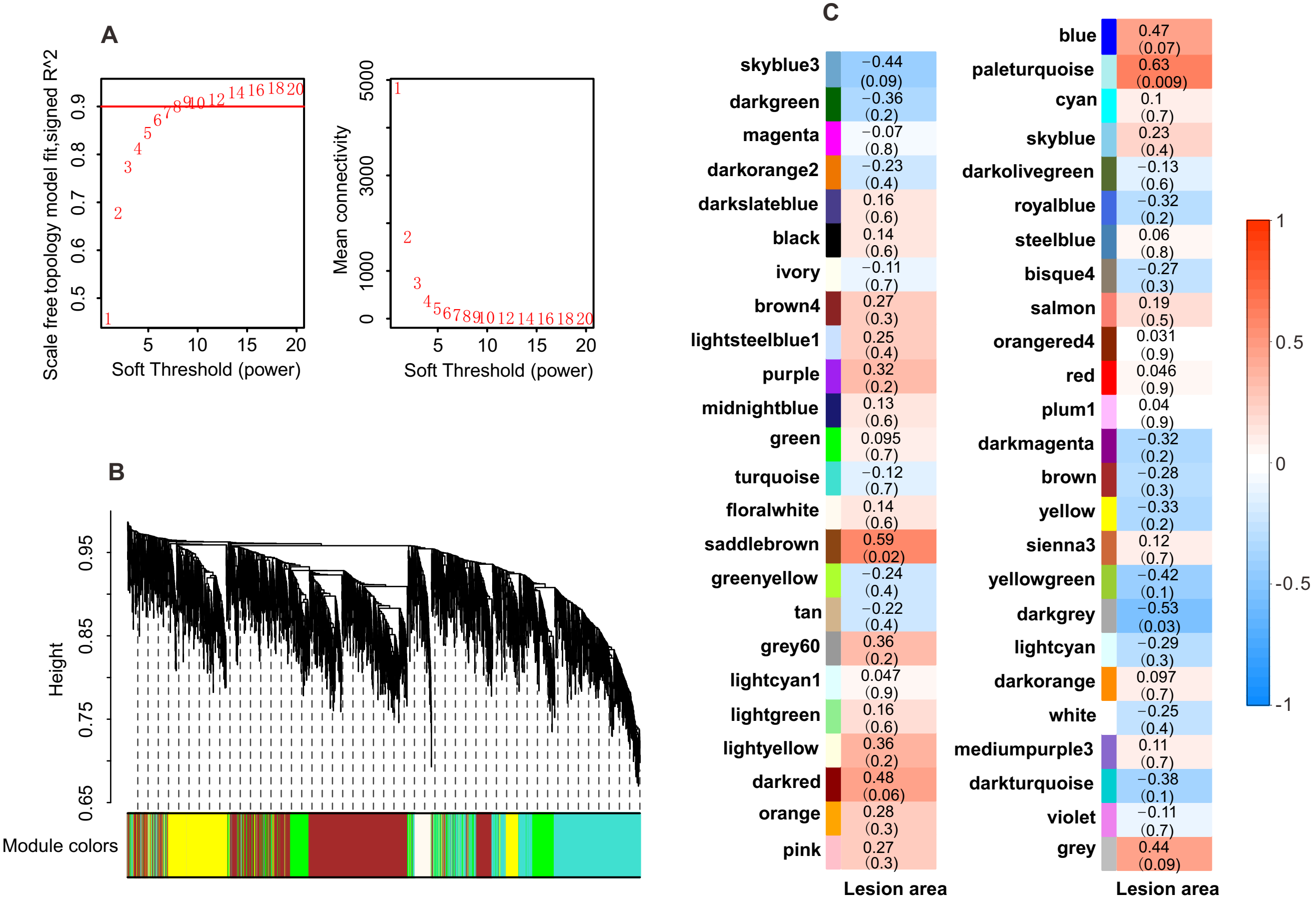

Supplementary Figure 1. Weighted gene co-expression network analysis (WGCNA) in HS and HSpm samples. **(A)** Analysis of network topology for various soft-thresholding powers. Panel (left) shows the scale-free fit index as a function of the soft-thresholding power. Panel (right) shows the mean connectivity as a function of the soft-thresholding power. **(B)** Clustering dendrogram of genes with dissimilarity based on topological overlap, together with assigned module colors. **(C)** Module-trait relationships. Each cell contains the corresponding correlation and *p* value (in parentheses).
